# Regional Differences in the Psychological Recovery of Christchurch Residents Following the 2010/2011 Earthquakes: A Longitudinal Study

**DOI:** 10.1371/journal.pone.0124278

**Published:** 2015-05-01

**Authors:** Lara M. Greaves, Petar Milojev, Yanshu Huang, Samantha Stronge, Danny Osborne, Joseph Bulbulia, Michael Grimshaw, Chris G. Sibley

**Affiliations:** 1 University of Auckland, Auckland, New Zealand; 2 Victoria University of Wellington, Wellington, New Zealand; 3 University of Canterbury, Christchurch, New Zealand; University of New South Wales, AUSTRALIA

## Abstract

We examined changes in psychological distress experienced by residents of Christchurch following two catastrophic earthquakes in late 2010 and early 2011, using data from the New Zealand Attitudes and Values Study (NZAVS), a national probability panel study of New Zealand adults. Analyses focused on the 267 participants (172 women, 95 men) who were living in central Christchurch in 2009 (i.e., before the Christchurch earthquakes), and who also provided complete responses to our yearly panel questionnaire conducted in late 2010 (largely between the two major earthquakes), late 2011, and late 2012. Levels of psychological distress were similar across the different regions of central Christchurch immediately following the September 2010 earthquake, and remained comparable across regions in 2011. By late 2012, however, average levels of psychological distress in the regions had diverged as a function of the amount of property damage experienced within each given region. Specifically, participants in the least damaged region (i.e., the Fendalton-Waimairi and Riccarton-Wigram wards) experienced greater drops in psychological distress than did those in the moderately damaged region (i.e., across the Spreydon-Heathcote and Hagley-Ferrymead wards). However, the level of psychological distress reported by participants in the most damaged region (i.e., across Shirley-Papanui and Burwood-Pegasus) were not significantly different to those in the least damaged region of central Christchurch. These findings suggest that different patterns of psychological recovery emerged across the different regions of Christchurch, with the moderately damaged region faring the worst, but only after the initial shock of the destruction had passed.

## Introduction

Since September 2010, two major earthquakes and nearly fifteen thousand aftershocks have struck the Canterbury region, which contains Christchurch, New Zealand’s third largest city [1, 2]. The first major earthquake occurred early in the morning of September 4^th^, 2010, and measured 7.1 on the Richter scale; this earthquake caused major structural damage, but thankfully claimed no lives. The Canterbury region then faced numerous challenges such as rebuilding a community affected by constant aftershocks and soil liquefaction [2–5]. Just as Cantabrians were beginning the process of reconstructing their city, a second major earthquake struck at 12:51pm on February 22, 2011. This earthquake not only caused further damage to the region (i.e., at least an estimated NZ$11 billion), but also claimed 185 lives [1, 2]. In the years that have passed since these major earthquakes, Cantabrians have been set the task of rebuilding not only their infrastructure, but also their mental health and wellbeing.

Unsurprisingly, natural disasters tend to have a negative effect on survivors’ mental health. Indeed, many studies show higher rates of Post-Traumatic Stress Disorder (PTSD) present in the population following a natural disaster, when compared with unaffected regions, and pre-disaster rates [6–9]. Earthquakes may present affected communities with further challenges as survivors not only have to contend with the trauma of a sudden natural disaster, and perhaps the associated displacement, loss of key services, financial security and damage to property, but also numerous aftershocks that make it difficult for psychological recovery and the rebuilding process to take place [3, 10, 11]. Indeed, research with earthquake survivors has shown higher levels of depression, anxiety, PTSD and stress in the affected population [9, 11–17]. Additionally, there is evidence that in the years following a disaster, stress levels may be maintained or even exacerbated as a result of dealing with insurance claims and other bureaucratic institutions that may sometimes be perceived as less than helpful [10, 11].

Research from Canterbury in the aftermath of the two major earthquakes has documented similar psychological consequences. Such findings include reports of intense fear, sleep disturbances, cognitive dysfunction, anxiety, hyper-vigilance, uncertainty, guilt, PTSD, and the burden of attending to other more vulnerable members of the community [18–23]. Qualitative research also suggests that uncertainty, chronic stress and feelings of tiredness became ‘the new normal’ for those who remained in Christchurch after February 2011 [17–19].

Similar results have been found using longitudinal data from a study that was underway before the first earthquake (*n* = 142) [23]. These researchers assessed mental health and personality over three time points pre-earthquake and found a decrease in mental health after both the September 2010 and February 2011 earthquakes that was predicted by pre-earthquake levels of neuroticism, depression and self-control. Similarly, research has found that those with higher levels of emotional stability (i.e., the converse of neuroticism) experienced less of an increase in psychological distress after the earthquakes, and that emotional stability decreased post-earthquakes, suggesting a greater vulnerability to depression, anxiety and other negative mental health outcomes [24, 25].

Whereas the above-mentioned studies examined the consequences of exposure to the earthquakes across the *general Canterbury region*, there are reasons to believe that differential changes in mental health may occur within *specific areas* of Christchurch (i.e., those with differential rates of damage). After the September 2010 earthquake, a door-to-door survey was completed across two neighbourhoods which had similar demographic compositions but varied on the amount of damage and the extent to which residents were displaced from their homes following the disaster [26]. They found that at 8–10 weeks post-earthquake, the two communities had no difference in terms of acute stress symptoms. Residents of the community that suffered more damage during the earthquake, however, had higher depression and anxiety scores than did residents of the other neighbourhood. This suggests that, although negative mental health effects were present across neighbourhoods, those who experience more property damage had higher levels of anxiety and depression post-earthquakes.

Other studies also suggest that some areas of Christchurch may recover more quickly than others, depending on the relative level of initial damage to the neighbourhood [3, 11, 18]. Certain regions of Christchurch experienced greater damage to buildings and land [27, 28]. These differential amounts of damage may mean that some neighbourhoods (those that were the least damaged) recover from the earthquakes more quickly than other neighbourhoods. Data from the New Zealand Attitudes and Values Study (NZAVS), a national longitudinal panel study provides us with the opportunity to examine this possibility.

### Overview and hypotheses

**Fig 1:**
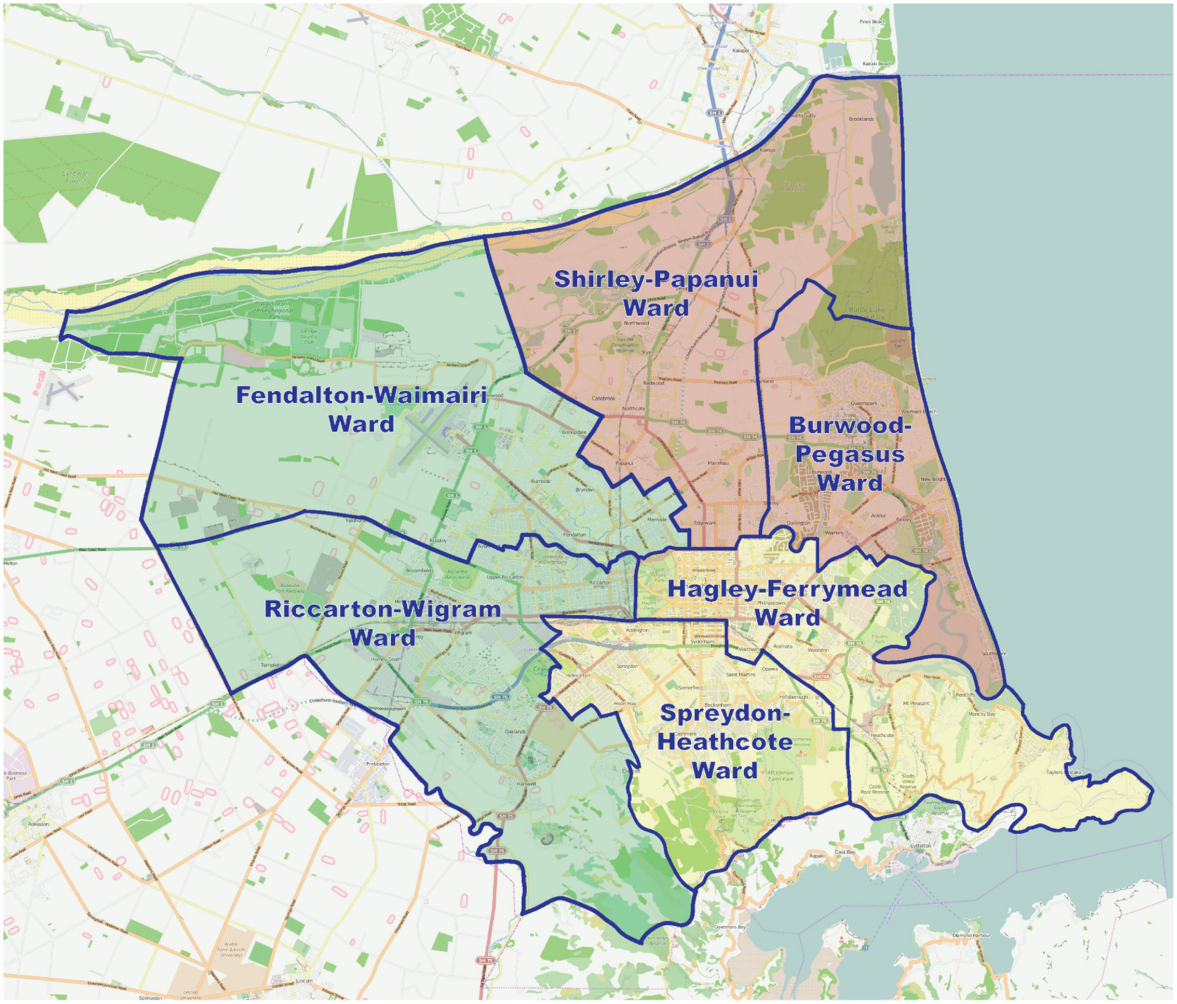
Map of the Christchurch region showing different wards and the different damaged-grouped regions. The most damaged wards were those in the North East (Shirley-Papanui and Burwood-Pegasus) followed by the moderately-damaged wards of Spreydon-Heathcote and Hagley-Ferrymead in the South-East, and the least-damaged wards of Fendalton-Waimairi and Riccarton-Wigram in the West. Note. Map was generated using http://www.openstreetmap.org.nz/.

The current study examines levels of non-specific psychological distress experienced by residents of Christchurch in a national probability panel study (i.e., the NZAVS). We focus our analyses on people who resided in the six wards that form central Christchurch and measure psychological distress using the Kessler-6 (K6) [29]. In order to ensure that we have an adequate sample size to test our hypothesis, participants were grouped together based on the level of damage experienced in their immediate neighbourhood. These groupings included the two most damaged wards (i.e., Shirley-Papanui and Burwood-Pegasus), the two moderately damaged wards (i.e., Spreydon-Heathcote and Hagley-Ferrymead) and the two (relatively) least-damaged wards (i.e., Fendalton-Waimairi and Riccarton-Wigram; see Fig 1 for the locations of these wards). In doing so, we compare scores on the K6 over these three regions of central Christchurch in late 2010, late 2011 and late 2012. The amount of damage experienced within a given ward (or grouping of wards) should correspond with rates of displacement and other psychological stressors (e.g., insurance claims, disruptions to daily life, etc.). Thus our hypothesis is that we predict K6 scores will remain highest in the areas that were most damaged by the earthquakes (i.e., Shirley-Papanui and Burwood-Pegasus), and that those in the least damaged region will show recovery, while the scores of those in the moderately-damaged region should be somewhere in between and show some degree of recovery.

## Method

The NZAVS is an ongoing 20-year longitudinal national probability study of social attitudes, personality and health outcomes that started in 2009. This paper analysed data from the NZAVS collected in 2010, 2011 and 2012 (the K6 measure was first included in 2010). In 2009, the NZAVS began by sampling a total of 6,518 New Zealanders from the New Zealand electoral roll. Here, we focus our analyses on the 267 participants (172 women, 95 men) whom we sampled in 2009 (i.e., before the Christchurch earthquakes) who were residing in the six wards that form central Christchurch, and who also provided complete responses to our yearly questionnaires conducted in 2010 (mostly completed after the September 2010 earthquake but before the February 2011 earthquake), 2011 (after the February 2011 earthquake) and 2012 (i.e., Wave 2 to Wave 4 of the NZAVS). Detailed information about the sample procedures, overall retention rates, demographic characteristics, and items included in the NZAVS questionnaires are provided on the NZAVS website [30–33].

### Regions

The six regions of Christchurch were operationalised by using 2008 classification codes from Statistics New Zealand to code the wards in which people resided when sampled for the first and only pre-earthquake wave of the NZAVS (i.e., 2009). Wards represent a mid-level classification separating New Zealand into 285 distinct regions. According to Statistics New Zealand the classification of wards is “designed to allow for the recognition of communities within a district and to increase community involvement in the local government system [34].” Based on the building damage ratio calculations from the Canterbury Earthquake Recovery Authority (CERA), we grouped together the six wards that form Central Christchurch based on the amount of relative damage suffered in the given ward [27, 28]. The most damaged (*n* = 80) were the Shirley-Papanui and Burwood-Pegasus wards, the moderately damaged (*n* = 91) were the Spreydon-Heathcote and Hagley-Ferrymead wards, and the least-damaged (*n* = 96) were the Fendalton-Waimairi and Riccarton-Wigram wards. The geographic boundaries for these wards are presented in Fig 1.

### Sampling procedure and retention rates

To provide a general overview of sample retention in Christchurch, initially (i.e., in 2009), we sampled between 80–100 people from each of the six wards that comprise central Christchurch (575 people in total), and retained between 51–66 participants from each of these wards in 2010 (i.e., the first wave of the study to include measures of psychological distress). The total retention rate from the pre-Earthquake 2009 wave in 2010 was 61.39%. As with the overall NZAVS, we had a gender bias, with women (*n* = 337) being more likely to respond than men (*n* = 238). In total, by 2012, we retained 267 participants (172 women, 95 men), who had provided age and gender and who completed the K6 in all three of these waves (a 75.64% retention rate from 2010). Research on the entire NZAVS over four time points has shown that men were more difficult to retain [35].

Critically, chi-square tests indicated that there were no differences in retention rate across the regions of focus from the initial sample in 2009 to 2010 (χ^2^(2,575) = 3.53, *p* = .171). However, there were differences in retention rate across regions from 2009 to 2011 (the first time point after the second earthquake; χ^2^(2, 575) = 8.36, *p* = .015), as the most damaged regions had a slightly lower retention rate (52.7%) than did the moderately damaged (61.3%) and least damaged (67%) regions. There were no differences in retention from 2009 to 2012 (χ^2^(2,575) = 5.03, *p* = .081). Furthermore, there were no differences in 2010 K6 scores between those who were retained and those who dropped out of the study in 2011 (*F*(1,349) = .443, *p* = .273 *partial η* ^*2*^ = .003) or in 2012 (*F*(1,349) = .690, *p* = .172, *partial η* ^*2*^ = .005). This is important because it indicates that bias in retention rates across regions is unlikely to account for the regional differences in K6 scores that we examine in 2010–2012.

### Questionnaire measures

Psychological distress in 2010, 2011 and 2012 was measured using the K6 scale developed by Kessler and colleagues [29]. The questions are shown in Table 1. The K6 scale summed scores for participants (both raw and covariate-adjusted) across wards are shown in Table 2. Individual K6 scores were calculated as totals in keeping with past research [29, 36,37]. The K6 is regularly used in the New Zealand Health Survey, and has also been validated for use in the NZAVS [36, 37]. The scale shows good item response properties and internal reliability when administered as part of the broader NZAVS questionnaire (scale properties, including sample means, can be found in Krynen, Osborne, Duck, Houkamau and Sibley, 2013) [36].

**Table 1:** Items of the Kessler-6 (K6) Non-Specific Psychological Distress Scales, scored on a 0 (none of the time) to 4 (all of the time) scale.

During the last 30 days, how often did…
… you feel nervous
… you feel hopeless?
… you feel restless or fidgety?
… you feel so depressed that nothing could cheer you up?
… you feel that everything was an effort?
… you feel worthless?

**Table 2:** Mean scores for the K6 in the different damage-grouped regions of Christchurch for the participants who completed the 2010, 2011 and 2012 NZAVS questionnaire.

		2010		2011		2012	
	N	M	SE	M	SE	M	SE
**Raw scores**							
Most Damaged	80	4.510	0.388	4.800	0.397	4.693	0.381
Moderately Damaged	92	4.848	0.362	4.891	0.371	5.028	0.355
Least Damaged	96	4.958	0.354	4.860	0.363	3.860	0.348
**Covariate-adjusted scores**							
Most Damaged	80	4.520	0.368	4.802	0.384	4.697	0.381
Moderately Damaged	91	4.777	0.345	4.752	0.359	4.967	0.357
Least Damaged	96	5.017	0.335	4.907	0.348	3.884	0.346

**Fig 2:**
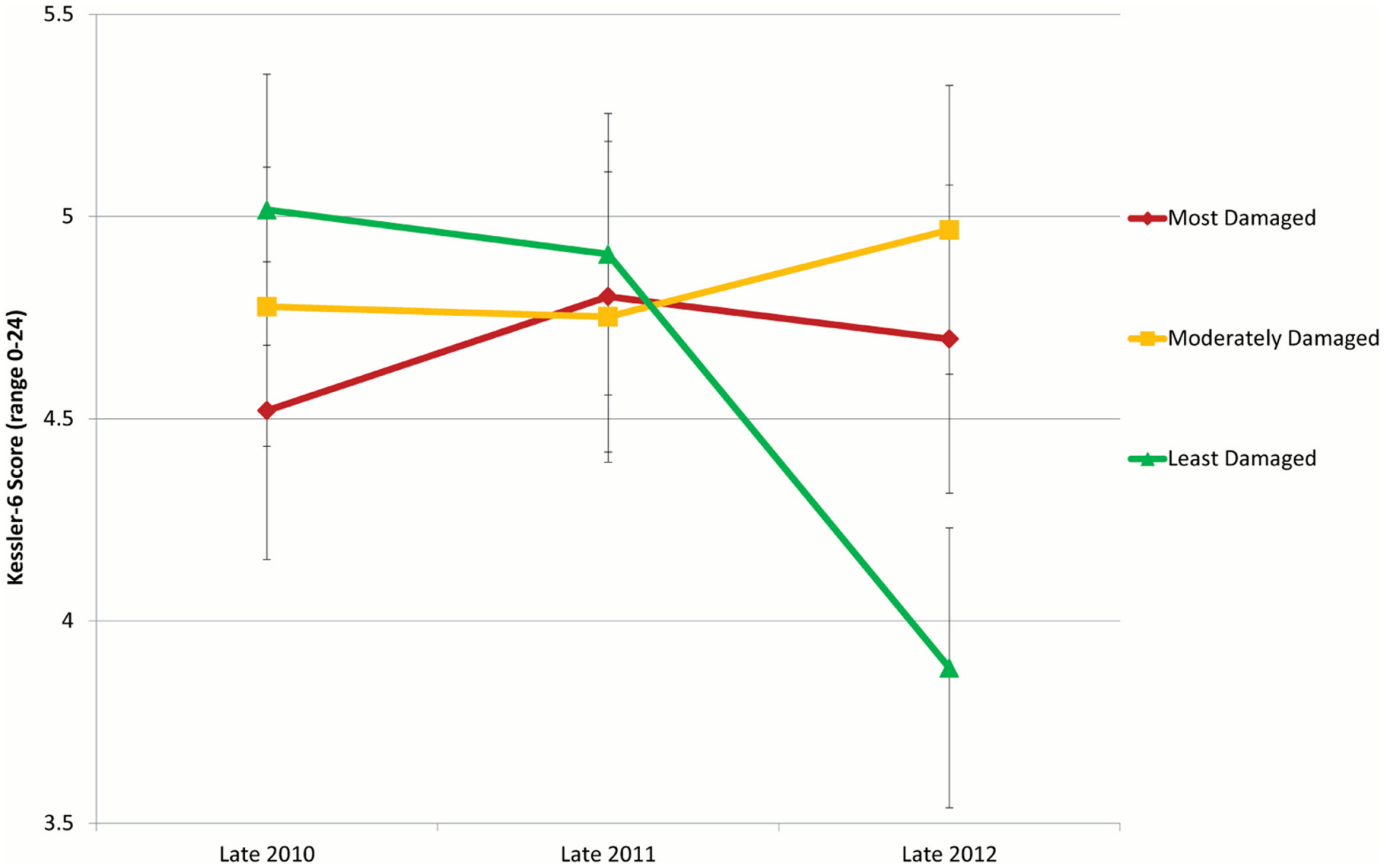
Kessler-6 scores across time for the three damaged-grouped regions in central Christchurch (means represent intercepts adjusting for gender and age, error bars represent the standard error of the intercept). Kessler-6 scores ranged from 0–24, with higher scores representing increased levels of psychological distress. The first of the large Earthquakes occurred in September 2011, the second in February 2012.

Adjusted scores included gender and age as covariates. Adjusted scores are graphed in Fig 2. N = 267.

### Ethics statement

The data reported in this study were collected as part of the NZAVS, which was approved by The University of Auckland Human Participants Ethics Committee. All participants gave written consent. Participants provided consent when completing the questionnaire, in their own time, and in their own space. The study was conducted in accordance with the principles expressed in the Declaration of Helsinki.

### Analytic approach

To examine our hypothesis that K6 scores changed over time as a function of the wards in which participants’ resided, we conducted a 3 (Time: 2010, 2011, 2012) × 3 (Regional Damage: Low, Moderate, High) repeated-measures ANCOVA on K6 scores using regional damage as a between-participants variable. Due to the type of analysis only those with complete responses in 2010, 2011, and 2012 were included in the analysis. Initial screening of the data indicated that the K6 scores approximated a normal distribution.

## Results

Gender and age were included as covariates in the analysis in order to adjust for possible gender and age differences across regions. Although not the focus of our analyses, the main effect of gender was not significant (*F*(1,262) = 2.361, *p* = .126, *partial η* ^*2*^ = .009), whereas there was a significant main effect of age (*F*(1,262) = 20.629, *p* <.001, *partial η* ^*2*^ = .073). Results also indicated that there was a significant main effect of time on K6 scores when averaged across regions (*F*(2,524) = 8.436, *p* < .001, *partial η* ^*2*^ = .031). The main effect of regional damage on K6 scores was non-significant (*F*(2,262) = .154, *p* = .857, *partial η* ^*2*^ = .001), indicating that, when averaged across the three time points, the levels of psychological distress did not vary across the three-levels of regional damage experienced in Christchurch.

However, our analyses yielded the predicted interaction between level of regional damage and time on K6 scores (*F*(4,524) = 3.458, *p* = .008, *partial η* ^*2*^ = .026). Though it is important to note that the interaction effect was fairly subtle (i.e., we explained 2.6% of the variance in K6 scores across time), we conducted a series of planned contrasts to examine our hypothesis that rates of psychological recovery would be hindered by the amount of damage experienced in the given region.

Results indicated that levels of psychological distress did not vary across regions in either 2010 or 2011. In 2012, however, the least damaged region (*M* = 3.860, *SD* = 2.725) had significantly lower K6 scores than the moderately damaged region (*M* = 4.996, *SD* = 3.916; *F*(1,262) = 4.753, *p* = .030, *partial η* ^*2*^ = .018). The difference between the least damaged and most damaged region (M = 4.693, SD = 3.531) was not significant, however (*F(*1,262) = 2.492, *p* = .116, *partial η* ^*2*^ = .009). Likewise, the planned contrast between the moderately damaged and most damaged region was also not significant (*F*(1,262) = .266, *p* = .606, *partial η* ^*2*^ = .001).

A series of Chi-Square tests were conducted to assess whether there were differences in the number of people suffering from mild/moderate or serious psychological distress across regions at each time point. Mild or moderate psychological distress is categorised as a score of over 8, whereas serious psychological distress is indicated by a score of 13 or more [29, 36,37]. There were no significant differences in 2010 (χ² (2) = 1.649, p = .438), 2011 (χ² (2) = 1.120, p = .571), or 2012 (χ² (2) = 2.395, p = .302) in the number of people suffering from a clinical level of psychological distress across region. Additionally, in 2010, 49 participants of the 267 reported at least mild distress, 53 in 2011 and 43 in 2012. Levels of psychological distress across the 3 regions and the 3 time points are presented in Fig 2. In sum, our analyses indicate that in 2012, a couple of years after the Christchurch earthquakes had occurred and recovery was well underway, it was people who lived in regions that initially received moderate levels of damage reported higher levels of psychological distress than those in areas that had been more lightly damaged.

## Discussion

The current study analysed data from participants who were living in the Christchurch region before two major earthquakes, and who completed a standardized (and well-validated) measure of psychological distress in late 2010 (largely between earthquakes), late 2011 (after the earthquakes) and again in late 2012 (approximately 2 years after the first earthquake). Specifically, we compared the levels of psychological distress reported by participants in this longitudinal panel sample over time and between three different damaged-based groupings of the six different wards that form central Christchurch. In doing so, we examined the possibility that levels of psychological distress may have decreased for some regions, but not others (i.e, those regions that suffered the most property damage).

On a positive note the mean total K6 scores across regions indicate a fairly low level of psychological distress in the population of central Christchurch. Indeed, the K6 is a DSM-IV diagnostic tool and, as such, is designed to screen for serious/clinical levels of mental distress [29, 36,37]. Because it is unlikely that a natural disaster would lead substantial portions of a population to develop a serious mental disorder, the relatively low mean levels of psychological distress reported in the current study are unsurprising.

### The least-damaged region

Our findings do, however, indicate that there are differential rates of psychological recovery among people living in the different regions of central Christchurch. These results corroborate previous research showing that those in the regions of Christchurch with the least amount of property damage reported the lowest levels of psychological distress [26]. Where we diverge from this earlier research is that we show that differences in recovery rates emerge *years* down the track [26]. Indeed, the two most damaged ward-groupings had fairly stable K6 scores over the time period, whereas the least damaged region (i.e., Fendalton-Waimairi and Riccarton Wigram) experienced a considerable drop in psychological distress in 2012. This suggests that the participants in the least-damaged region had some level of psychological recovery. Although we do not know the participant’s pre-earthquake K6 scores, our results show a decrease in scores in 2012 since the initial data collection, that is, around the time of the first big earthquakes, and following data collection in 2011, the year after the earthquakes.

### The moderately-damaged and most-damaged regions

In late 2012, the level of psychological distress reported by those who had lived (or were continuing to live) in the moderately-damaged wards of Hagley-Ferrymead and Spreydon-Heathcote was significantly higher than the levels of psychological distress reported by those who had lived (or were continuing to live) in the (least-damaged) region covered by the Riccarton-Wigram and Fendalton-Waimairi wards. In other words, participants living in the moderately-damaged regions of Christchurch failed to experience a drop in psychological distress nearly three years after the two major earthquakes that struck the Canterbury region.

Although we hypothesised that those from the moderately-damaged region would show some level of psychological recovery, they did not. One plausible reason for the lack of recovery among those living in this region may be that the earthquakes also altered the geology of the Heathcote River (which runs through the Spreydon-Heathcote ward). As such, residents were subjected to increased risks of flooding following the earthquakes [38]. Indeed, the Heathcote River has ‘burst its banks’ many times over the last couple of years following the two major earthquakes, which has resulted in further damage—especially in light of the earthquake-damaged drainage system—and additional insurance claims for its residents. This may have slowed psychological recovery in this region.

It is interesting to note that respondents in the most- and least-damaged regions of central Christchurch reported comparable levels of psychological distress throughout 2010 and 2011. It may be that the unprecedented amount of government support that emerged following the catastrophic damage experienced by residents within the most-damaged region mitigated many of the adverse long-term psychological effects of the earthquakes. Consistent with this possibility, in a rare systematic investigation of post-disaster recovery, researchers looked at rates of psychological recovery following a natural disaster in two Chinese villages of similar socioeconomic status, but that experienced different levels of initial damage and post-earthquake support [17]. Specifically, a massive amount of government support was given to the village that experienced the most destruction. Although the village with the most damage initially reported higher levels of psychological distress, respondents from this village rebounded on measures of quality of life and psychological wellbeing to a far greater extent than did those from the village that experienced relatively less destruction (and received less government support). This suggests that the amount of support provided to a disaster-affected community can greatly mitigate the psychological toll of experiencing a natural disaster. Whether or not this process occurred within our sample is an important question that should be addressed in future research, but our findings are suggestive.

### Future research directions and limitations

The data reported here represent one of the only longitudinal panel studies examining change in the psychological outcomes of Christchurch residents following the earthquakes. Nevertheless, it is critical to keep in mind that our analyses examined *average* levels of change in psychological distress across regions over time. As such, rather than examining change within individuals, our analyses inform us about aggregate changes in the overall level of psychological distress among people in the region over time.

Although such an approach provides us with valuable information on the average differences of psychological recovery across regions, our analyses do not speak to the possibility that some *individuals* may have experienced larger changes in psychological distress than did others. Thus, it is possible that some people may have experienced a decrease, whereas others may have experienced an increase, in psychological distress over time. The ability to examine these questions about what might predict some people experiencing more change than others over time requires the use of latent growth models. We are eagerly awaiting the collection of future waves of the NZAVS so that we can examine the predictors of individual differences in the rate and trajectory of change following the Christchurch earthquakes.

It should also be noted that we do not know what the pre-earthquake K6 scores were for participants, as unfortunately the K6 measure was not included in the 2009 wave of the study. Therefore we are assuming that where our results show a decrease in K6 scores for the least damaged region, this *indicates* recovery over the time period. Extant research shows that natural disasters tend to cause psychological distress, especially an increase in those suffering from various mental illnesses [3, 6–11, 17]. As such, we cannot confirm that the people in the least damaged region have returned to pre-earthquake levels of psychological distress. Rather it suggests that psychological distress has decreased in the years following the earthquakes. Again, more time points are needed to come to firmer conclusions about the recovery process.

Moreover, we asked participants to report their levels of psychological distress using the K6 self-report measure, which is a well-validated, national population screening tool designed to detect psychological distress that may indicate risk of or the presence of mental illness [29, 36,37]. However, we believe it can also be helpful to detect change at lower levels of the scoring range. Any significant changes in the mean level of psychological distress, even one or two points on the aggregate, indicate a meaningful increased risk in the population (as this could mean the difference between a number of people being of ‘no psychological distress’ instead of ‘moderate psychological distress’).

Additionally, using the K6 over the period of a month provides a narrower ‘snapshot’ of one’s mental health, versus asking participants to reflect on the past year, which may have provided a broader look at their mental health over the annual survey period. While we opted for using the 30-day version of the measure in our surveys in order to secure discrete assessments over time, potential consequences of this approach must be acknowledged. Assessing K6 scores on a per year basis would possibly have not shown the beginning of the psychological recovery for participants in the least-damaged region, as our results have shown. It may be that the differences in recovery occurred more recently than the past year for participants, Participants who completed the survey in late 2012, would be including a period of aftershocks in their appraisal. The overall time-line for recovery is something that we aim to follow-up on as more time points become available.

Another interesting direction for future research is to examine the effects of participants’ migration patterns following the earthquakes on their rates of recovery. It is possible that there are critical differences in psychological distress between those who a) stayed in their original houses and wards, b) moved wards within the Canterbury region, and c) moved away from the region altogether. Given that the current paper demonstrated that the amount of damage within the region influenced people’s recovery rates, it is likely that there will be critical differences between these groups on important mental health outcomes. Future research should examine this possibility, as well as the likelihood that individual differences (e.g., personality, resilience, or connection with the community) and demographic variables (e.g., socio-economic status) predict whether or not one remained in the Canterbury region/Christchurch post-earthquakes.

### Concluding comments

Our findings indicate that there are differential rates of psychological recovery among people living in the different regions of central Christchurch. Specifically, recovery varied as a function of the initial amount of property damage caused by the Christchurch earthquakes. On a positive note, our findings show that the K6 scores obtained in the study for most respondents were in the range of ‘no psychological distress’, according to the standard K6 scoring procedure [29, 36,37]. Nevertheless, our results demonstrated that there was a disparity across regions in terms of psychological recovery. We suspect that this will not come as a surprise to many people living in the Canterbury region. Our informal impression is that these differences in psychological outcomes are mentioned fairly regularly within the Christchurch community. Our results add to this discussion by quantifying the magnitude of these effects and identifying the areas that are particularly affected. Such an approach is important as it provides critical insights into the areas that most need assistance during the recovery process and those who may *still* need assistance for their recovery.
